# Ischemic hepatitis due to an occlusion of visceral arteries: a case report

**DOI:** 10.1093/jscr/rjad671

**Published:** 2023-12-16

**Authors:** Safwan Omran, Andreas Greiner

**Affiliations:** Charité – Universitätsmedizin Berlin, corporate member of Freie Universität Berlin and Humboldt-Universität zu Berlin, Department of Vascular Surgery, Hindenburgdamm 30, 12203 Berlin, Germany; Charité – Universitätsmedizin Berlin, corporate member of Freie Universität Berlin and Humboldt-Universität zu Berlin, Department of Vascular Surgery, Hindenburgdamm 30, 12203 Berlin, Germany

**Keywords:** ischemic hepatitis, chronic mesenteric ischemia, mesenteric artery occlusion, visceral reconstruction

## Abstract

Ischemic hepatitis due to mesenteric artery occlusion is extremely rare. This is due to the function of the collateral network of the celiac–mesenteric arterial system and portal venous flow. A 64-year-old male presented with abdominal pain, a significantly reduced general condition, a weight loss of 20 kg in 4 months. Computed tomography showed occlusion of the celiac trunk and the superior mesenteric artery and hypodense lesions in the liver. We performed an antegrade visceral reconstruction with a bifurcated 12-6 mm Dacron graft from the supra-celiac aortic donor to the superior mesenteric and celiac arteries. The postoperative course and follow-up were uneventful.

## Introduction

Ischemic hepatitis, also known as shock liver or hypoxic hepatitis, is a liver disease caused by reduced blood flow to the liver [1]. Causes of ischemic hepatitis include systemic hypotension, heart failure, shock, severe hypoxic respiratory failure, and sepsis. Although arterial complications are a known cause of liver transplant failure, liver infarction due to mesenteric artery occlusion is extremely rare [2]. Other reasons for the rarity of ischemic hepatitis are maintained portal venous blood flow and arterial collateralization [3]. In this article, atypical hypodense lesions in the liver were observed in the CT-angiography as a result of the occlusion of the celiac trunk and the superior mesenteric arteries.

## Case presentation

**Figure 1:**
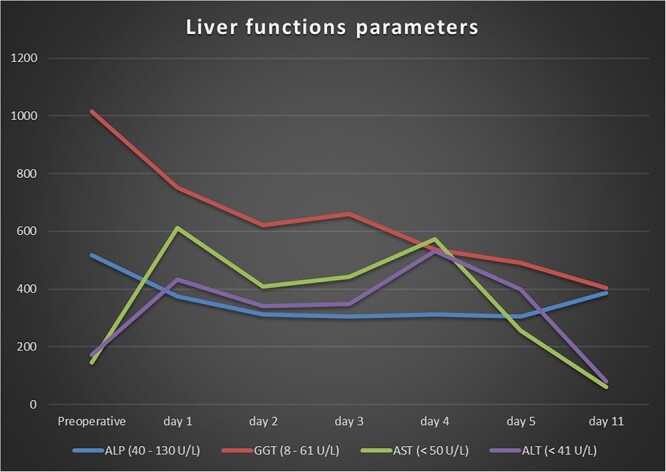
Laboratory values of the liver function parameters.

A 64-year-old male arrived at our central rescue center with abdominal pain and a significantly reduced general condition. Eating was not possible due to nausea and vomiting, and laboratory tests revealed increased liver function parameters and increased parameters of inflammation (Fig. 1). The patient had already been diagnosed in an external hospital with a weight loss of 20 kg in 4 months and was suspected of having chronic mesenteric ischemia. The patient’s weight was 60 kg, height 179 cm, and body-mass-index 18.7. Preexisting conditions included hypertension, left posterior cerebral artery stroke, and left basal ganglia stroke with facial paralysis and dysarthria, antral gastritis with a history of hemorrhagic gastritis, intermittent atrial fibrillation, mild mitral and tricuspid regurgitation, nicotine abuse, alcohol abuse, and degenerative spine disease. Previous surgeries included cholecystectomy.

**Figure 2:**
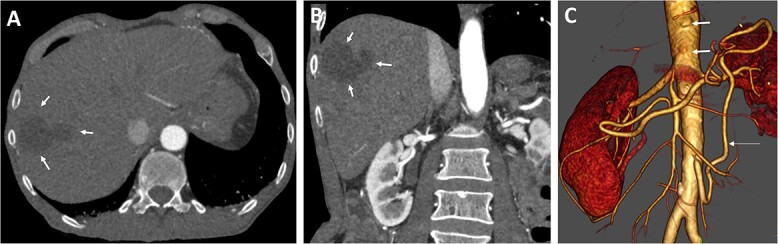
Preoperative axial (A) and coronal (B) CT angiographic images demonstrate hypoenhancement of all of the hepatic parenchyma and hypodense, hepatic lesion measuring 48 × 47 × 34 mm in the segment V and VI (arrows). (C) 3D reconstructed CT angiography shows occlusion of the celiac and superior mesenteric arteries (thick arrows) and hypertrophy of the inferior mesenteric artery and artery of Drummond (thin arrow).

Various antibiotics were started, including meropenem 1 g every 8 hours, vancomycin 1 g every 12 hours, and cefuroxime 500 mg every 8 hours. Computed tomography performed showed occlusion of the celiac trunk and the superior mesenteric artery with prominent collateralization through the inferior mesenteric artery (Fig. 2). The intrahepatic portal vein was patent. In addition, hypodense lesions were found in the liver, which most closely corresponded to hepatic ischemia. The indication for surgical reconstruction was made. We performed an antegrade visceral reconstruction with a bifurcated 12-6 mm Dacron graft from the supra-celiac aortic donor to the superior mesenteric and celiac arteries without any complications.

**Figure 3:**
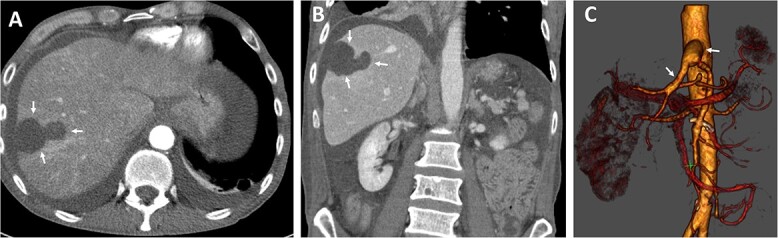
Postoperative axial (A) and coronal (B) CT angiographic images demonstrate enhancement during contrast-enhanced phase and hepatic ischemic area with irregular margins. (C) 3D reconstructed CT angiography shows antegrade visceral reconstruction with a bifurcated graft from the supra-celiac aortic donor to the superior mesenteric and celiac arteries (arrows).

The postoperative recovery was uneventful. We achieved adequate pain control with patient-controlled intravenous analgesia. Inflammation and transaminases levels decreased, and symptoms significantly improved. Diuresis was adequate with normal renal parameters. We performed Partial Thromboplastin Time (PTT)-controlled anticoagulation with a target PTT of 50–60 seconds using intravenous heparin. The patient was transferred to the normal departure on the third postoperative day. Postoperative CT angiography demonstrate enhancement during contrast-enhanced phase and hepatic ischemic area with irregular margins (Fig. 3).

**Figure 4:**
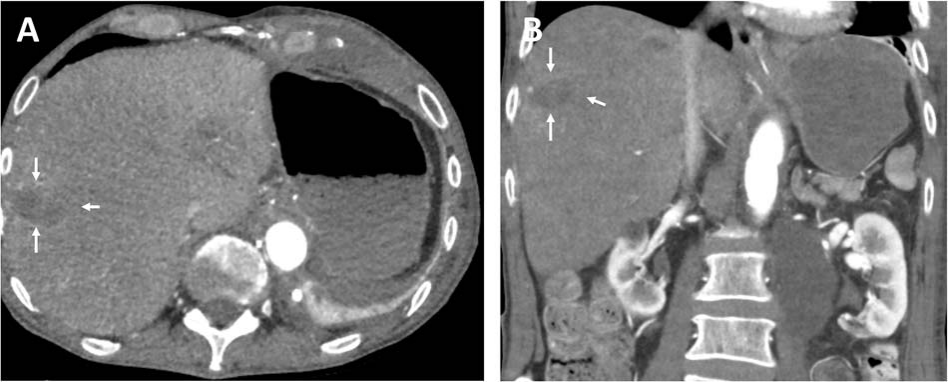
Follow-up (46 months after liver infarction) axial (A) and coronal (B) CT angiographic images demonstrate a stable hepatic infarction lesion 33 × 18 × 20 mm (arrows).

The oral antibiotics were administered and continued for another 10 days, and the patient was discharged on the 12th postoperative day in good general condition, fully mobilized, free of fever and infections. Follow-up CT showed a stable hepatic lesion (Fig. 4).

## Discussion

Ischemic hepatitis is a condition in which blood flow and consequently oxygen delivery to the liver is reduced, leading to liver damage [1, 4]. Reports of ischemic hepatitis due to mesenteric artery occlusion are extremely rare. This is due to the function of the collateral network of the celiac–mesenteric arterial system and portal venous flow [5].

Although ischemic hepatitis most often occurs after cardiac arrest, severe hypotension, shock, sepsis, or hepatic artery occlusion, there is scarce literature of ischemic hepatitis due to visceral arteries stenosis [1, 6–9].

The symptoms of ischemic hepatitis can vary depending on the severity of the liver injury. Mild cases may not present with any symptoms, while more severe cases can lead to liver failure. Some common symptoms of ischemic hepatitis include nausea and vomiting, abdominal pain, jaundice, fatigue and weakness, dark urine, clay-colored stools, and enlarged liver [10].

The diagnosis of ischemic hepatitis mainly depends on the liver biochemical tests to check liver function in clinical practice [1, 11, 12]. Imaging tests such as ultrasound, CT scan, or MRI may be used to evaluate the liver and surrounding organs [13]. A liver biopsy may also be performed to confirm the diagnosis and determine the severity of the liver damage.

The treatment of ischemic hepatitis depends on the underlying cause and severity of the liver injury. Mild cases may not require any treatment and can be managed with rest and monitoring. In more severe cases, hospitalization may be necessary. Treatment may include fluid replacement to maintain blood pressure and improve blood flow, medications to manage symptoms such as nausea and vomiting, nutritional support to help the liver recover, monitoring and management of complications such as liver failure or sepsis.

Prevention of ischemic hepatitis involves identifying and managing underlying conditions that can lead to decreased blood flow to the liver. This includes maintaining adequate fluid and electrolyte balance, treating underlying infections or sepsis, and managing heart failure or other conditions that can lead to shock. In the case of visceral artery stenosis, a reconstruction of the visceral arteries using endovascular or open surgical options may help to improve the blood flow in the liver. Whereas percutaneous angioplasty and stenting are the first choice for the treatment of mesenteric artery stenosis, when possible, surgical options remain for the treatment of complex pathologies and stenosis recurrence [14, 15].

In conclusion, ischemic hepatitis due to occlusion of all visceral arteries is a rare condition that can lead to liver injury and dysfunction due to decreased blood flow to the liver. It can lead to symptoms such as nausea, abdominal pain, and jaundice. Treatment depends on the severity of the liver injury and may involve reconstruction of the visceral arteries with a bypass.
